# Experimental characterization, modelling and compensation of temperature effects in optotunable lenses

**DOI:** 10.1038/s41598-023-28795-7

**Published:** 2023-01-28

**Authors:** Yassine Marrakchi, Xoana Barcala, Enrique Gambra, Ivan Martinez-Ibarburu, Carlos Dorronsoro, Lucie Sawides

**Affiliations:** 1 2EyesVision, Plaza de La Encina, 10, Nucleo 3, Planta 4, 28760 Tres Cantos, Madrid Spain; 2 Instituto de Optica, Consejo Superior de Investigaciones Cientificas, IO-CSIC, Serrano 121, 28006 Madrid, Spain

**Keywords:** Engineering, Optics and photonics

## Abstract

Most tunable lenses (TLs) are affected by deviations in optical power induced by external temperature changes or due to internal heating while in use. This study proposes: (1) An experimental characterization method to evaluate the magnitude of the optical power deviations due to internal temperature shifts; (2) three different mathematical models (experimental, polynomial, and optimized) to describe the response of the lens with temperature; (3) predictions of the internal temperature shifts while using the lens in time frames of minutes, seconds, and milliseconds and; (4) a real time optical power compensation tool based on the implementation of the models on a custom voltage electronic driver. The compensation methods were successfully applied to two TL samples in static and dynamic experiments and in hysteresis cycles. After 40 min at a static nominal power of 5 diopters (dpt), the internal temperature exponentially increased by 17 °C, producing an optical power deviation of 1.0 dpt (1.5 dpt when the lens cools down), representing a 20% distortion for heating and 30% for cooling. Modelling and compensation reduced the deviations to 0.2 dpt when heating (0.35 dpt when cooling) and the distortions to 4% and 7%. Similar levels of improvement were obtained in dynamic and hysteresis experiments. Compensation reduced temperature effects by more than 75%, representing a significant improvement in the performance of the lens.

## Introduction

The advantages of focus tunable lenses (TLs) over traditional fixed optics are clear-cut in several applications. Having the possibility of modifying the optical power or the zoom of an optical system with an electrical signal, without moving parts, provides a wide range of opportunities in optical design. These lenses can improve compactness, speed and stability by reducing the number of opto-mechanical components required to generate a shift on focus^1^. In recent years, the use of TLs has expanded to many application fields with considerable success. Some representative examples are autofocusing in imaging systems^2^ and traditional optical microscopy^3^. Zoom mechanisms in machine vision^4^ and similar configurations in medical imaging in endoscopes^5^ and laparoscopy^6^. Also, in the clinical environment, TLs provided interesting improvements in depth scan optical coherence tomography (OCT)^7^, refractive power correction in ophthalmology^8^ and pre-operative simulation of multifocal intraocular lens patterns^9^. In recent years, TLs are widely used in 3-D microscopy enabling more flexibility in axial scanning^10–12^. Finally, they have been used in optical manipulation of atomic structures^13,14^, laser beam adjustments^15^ and virtual reality^16^, among others.

There exist different kinds of TLs in terms of their design and technology. Many of them take advantage of the variable shape of liquids and a transparent interface that is usually an elastic membrane. The driving system may be an electromagnetic or mechanical actuator^1^, electrically controlled liquid crystals^17^, electrowetting^18^, dielectrophoresis-actuated^19^, stimulus-responsive hydrogels^20^, pneumatic actuators^21^ or acoustic generators of gradient index of refraction (GRIN)^22^. Other approaches are also available as the Moiré-lenses^23^ or variations of the Alvarez lens for miniature devices^24^. The advantages and limitations of each alternative make the choice dependent on the target application. In this study, we set the focus on the electromechanically driven liquid lenses.

The performance of TLs is critical for the correct functioning of the devices and instruments that rely on them. Their application requirements include stability over time, and speed to shift from a focus state to another, especially in rapid scanning applications^25^ and temporal multiplexing^26^. But more important are the precision and accuracy that they must achieve in terms of optical power, and the robustness against modifications of the internal or external environment conditions as temperature. Some specific lenses exploit the property of dielectric liquids to expand with an increase of temperature^27^. However, for most TLs available, internal or external temperature changes represent an issue inducing important instabilities and reducing the precision of the optical power delivered by the lens^28^. Driving a TL at medium or high currents, or due to absorption of high-power laser light, provoke an internal temperature raise^29^. These deviations are important and often not linear with the change in the temperature. As an example, in the lens model under study (10-30-C; Optotune AG, Switzerland), according to the specifications of the manufacturer, the optical power increases in average 0.07 D each 1 °C increment on temperature^29^, when the temperature is around 25 °C. Commercial lens manufacturers have developed some hardware strategies to decrease the magnitude of the deviations, but they are limited to small dioptric ranges^30,31^. On the other hand, software compensation based on characterization of individual lenses at different temperatures and interpolation, requires specific drivers and therefore has limited options in the design of the corrections.

With a series of experimental behavior characterizations of liquid optotunable lenses and computational mathematical models, we propose a versatile solution to predict and compensate, in real time, for the power deviations with temperature variations of electromechanically actuated optotunable lenses. Addressing a challenge that, if accomplished, would improve the TLs’ performance, open new applications and facilitate their adoption in industrial and daily life products.

## Results

Four different behavior characterization experiments, with increasing complexity, were performed on two optotunable lenses (Optotune AG, Switzerland) aiming at replicating the working conditions or specific requirements of optotunable lenses for different applications. First, during the static experiment (2.1), flat states of the optotunable lens were applied for 40 min to study the internal heating and cooling processes and develop three mathematical models to compensate for (and ultimately predict) the power deviations due to temperature variations. Those solutions were implemented in a real time algorithm and evaluated in a hysteresis experiment (2.2), where the optotunable lens was configured to mimic a scanning application with a stair pattern of different power states; a random experiment (2.3) where random power jumps were applied to the optotunable lens to mimic more exigent requirements for the lens; and lastly, a dynamic experiment (2.4) that reproduced the use of optotunable lens in a multifocal correction simulation. During all the tests the TL was driven by a PWM voltage signal that varied between 0 and 5 V by controlling its duty cycle with an 8-bit timer (0 ‘counts’ is the minimum value and 255 is the maximum). The optical power was measured with a custom focimetry system^32^ and the temperature was registered with the internal sensor encapsulated in the sample lens.

### Static experiment

**Figure 1:**
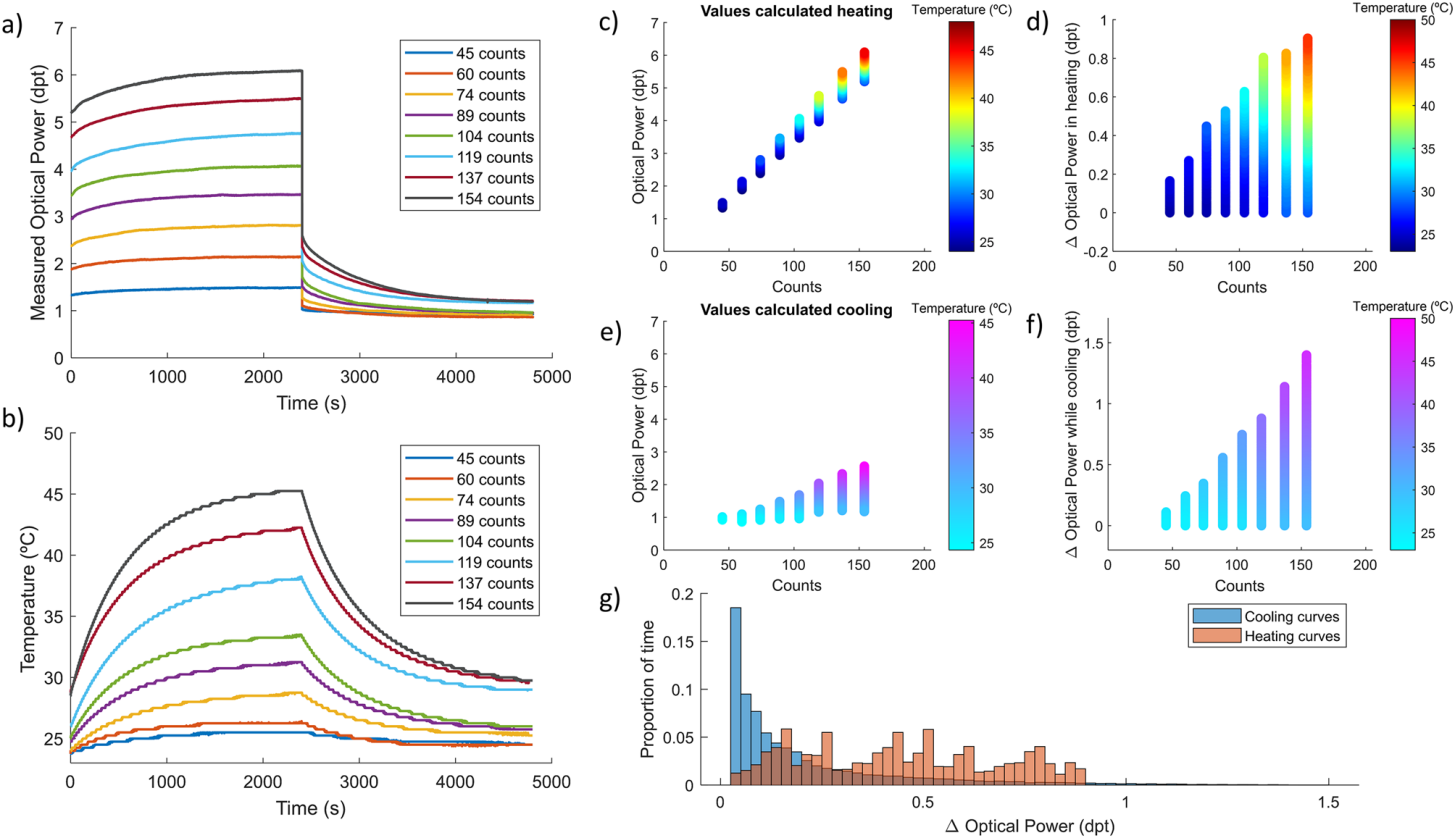
Characterization of temperature effect in a TL during long static states of different optical power. (**a**) Graphical description of the static experiment showing the optical power variation with time due to temperature variation. (**b**) Profile of the lens’ internal temperature during the experiment with different voltage driving signals. Measured optical power of the lens while heating (**c**) for different driving signals and cooling (**e**) at rest state. Estimated deviation, calculated from the difference between the initial point and measured optical power in the heating (**d**) and cooling (**f**) processes. (**g**) Histogram of optical power deviation while heating (red) and cooling (blue) to quantify the proportion of time the lens diverges a certain magnitude.

The static behavior of the TL was studied, and the temperature effect characterized to provide a reference for the development and evaluation of temperature compensation strategies. The lens was tuned to a specific flat signal state for 40 min to analyze the heating process due to current flowing and then suddenly released to a resting state to observe the cooling for another 40 min. The larger the electrical signal driving the lens, the higher is the optical power induced but also the deviations from a flat optical response due to temperature, as we can see in Fig. 1a. Moreover, as can be observed in Fig. 1b, during the heating (the first half of the experiment) the temperature increases faster, and points at a higher stability point, when the driving signal is higher. Figure 1c represents the measured optical powers of the lens for the different driving signals while heating and Fig. 1e while cooling at rest state. From that, we calculated the difference between the target value and the measured optical power to provide an objective metric evaluating the impact of the temperature in the heating (Fig. 1d) and cooling (Fig. 1f) processes. Finally, in Fig. 1g a histogram of optical power deviations while heating (orange) and cooling (blue) is plotted to show the proportion of time corresponding to each optical power deviation.

The behavior of the TL can be characterized through mathematical models in a 3-dimensional space of optical power, internal temperature and electrical signal to guide strategies for compensating the effect of temperature. Three models, that provide good fit to the characterization experiment data, were developed. (1) The optimized model that includes many parameters to provide the best fitting performance although diverging near the zero diopter. (2) The polynomial model (simpler than the optimized) based on a second-order polynomial equation in optical power and a first-order polynomial in temperature that provide good enough fitting factors while keeping the model simple, easy to understand physically and no divergence near zero diopter. (3) The experimental model based on simple calibrations of the driver relating optical power and voltage at a reference temperature with added temperature dependency parameters, optimized experimentally following a trial-and-error strategy (Sect.  “Mathematical models and compensation strategies”).

**Figure 2:**
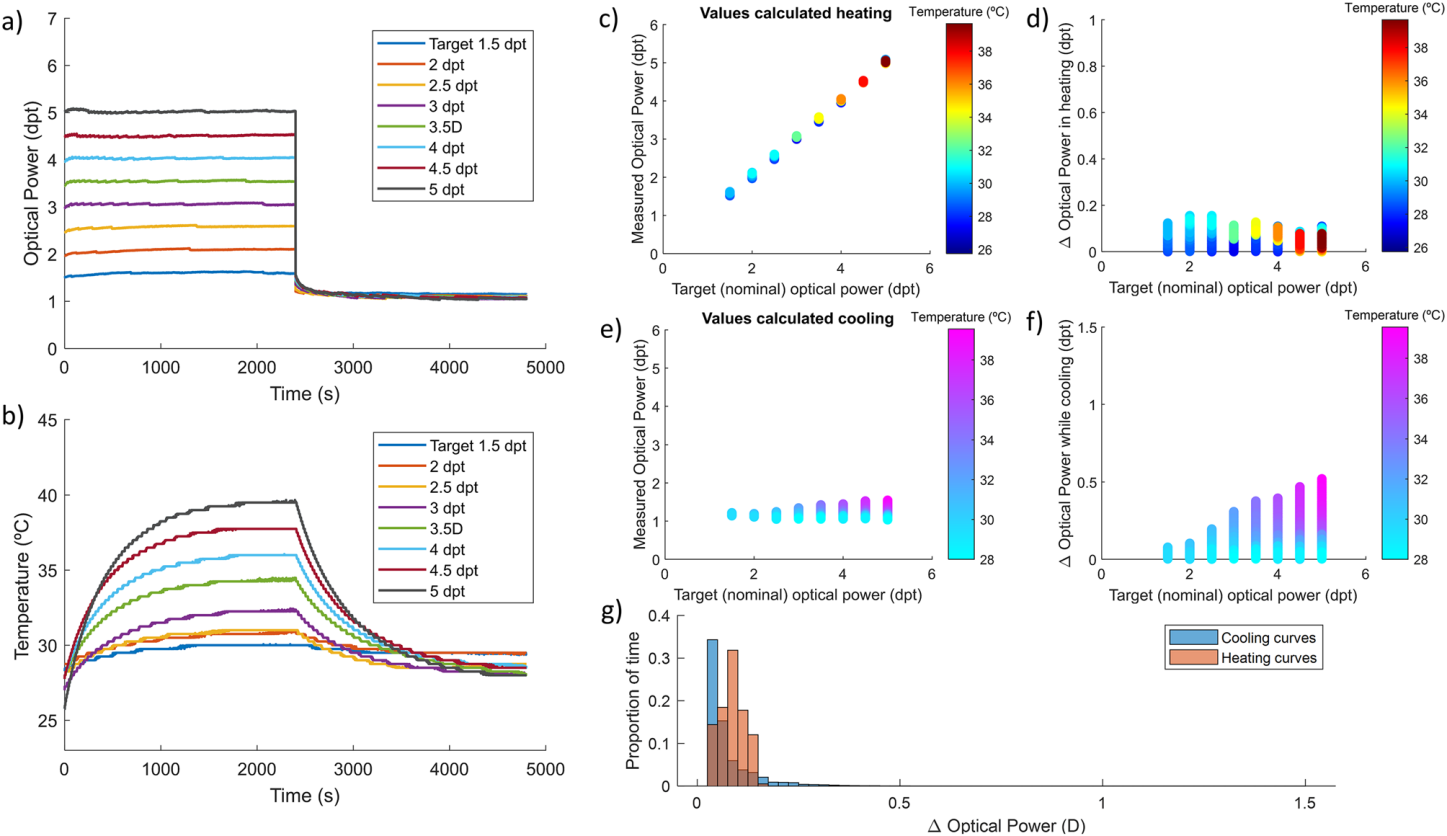
Optimized model compensation of temperature effect in a TL during long static states of different optical power. (**a**) Graphical description of the result of compensation strategy showing the optical power stabilization in time. (**b**) Internal temperature profile of the lens during the experiment with different target power states. Measured optical power of the lens while heating (**c**) for different driving signals and cooling (**e**) at rest state. Estimated deviation, calculated from the difference between target power and measured optical power in the heating (**d**) and cooling (**f**) processes. (**g**) Histogram of optical power deviation while heating (red) and cooling (blue).

Figure 2 shows the results obtained in the static experiment applying in real time the optimized fit model. We can observe that although the temperature increases with time Fig. 2b, the optical power is compensated by adjusting the electrical signal driving the TL. If we observe the curves of Fig. 2a carefully, we can see a sawtooth pattern that represent the small correction steps performed each time a variation in temperature of 0.5 °C is detected and the recalculation is performed by the software. The higher stability in the flat states is confirmed in Fig. 2c while heating and Fig. 2e while cooling since the measured optical power points distribution is narrower when the compensation is applied. The plots presenting the deviation between the target optical power and the measured one also demonstrate a great performance of the correction procedure in the heating process of the lens with less than 0.20 dpt deviation Fig. 2d against 1.00 dpt deviation for non-corrected measurements Fig. 1d. Concerning the cooling curves, the deviations are more important because, as described by the TL manufacturer and observed in the characterization Fig. 1f, temperature effect is more noticeable when driving the lens at lower currents. But if we observe the transition to home state in Fig. 2a and the histogram in Fig. 2g this only happens at the first few seconds after the lens is returned to the rest state. In any case, the deviation magnitudes remain smaller than for the non-corrected experiment: maximum value of 0.50 dpt applying the optimized model (Fig. 1f) versus 1.60 dpt when uncorrected (Fig. 2f).

**Figure 3:**
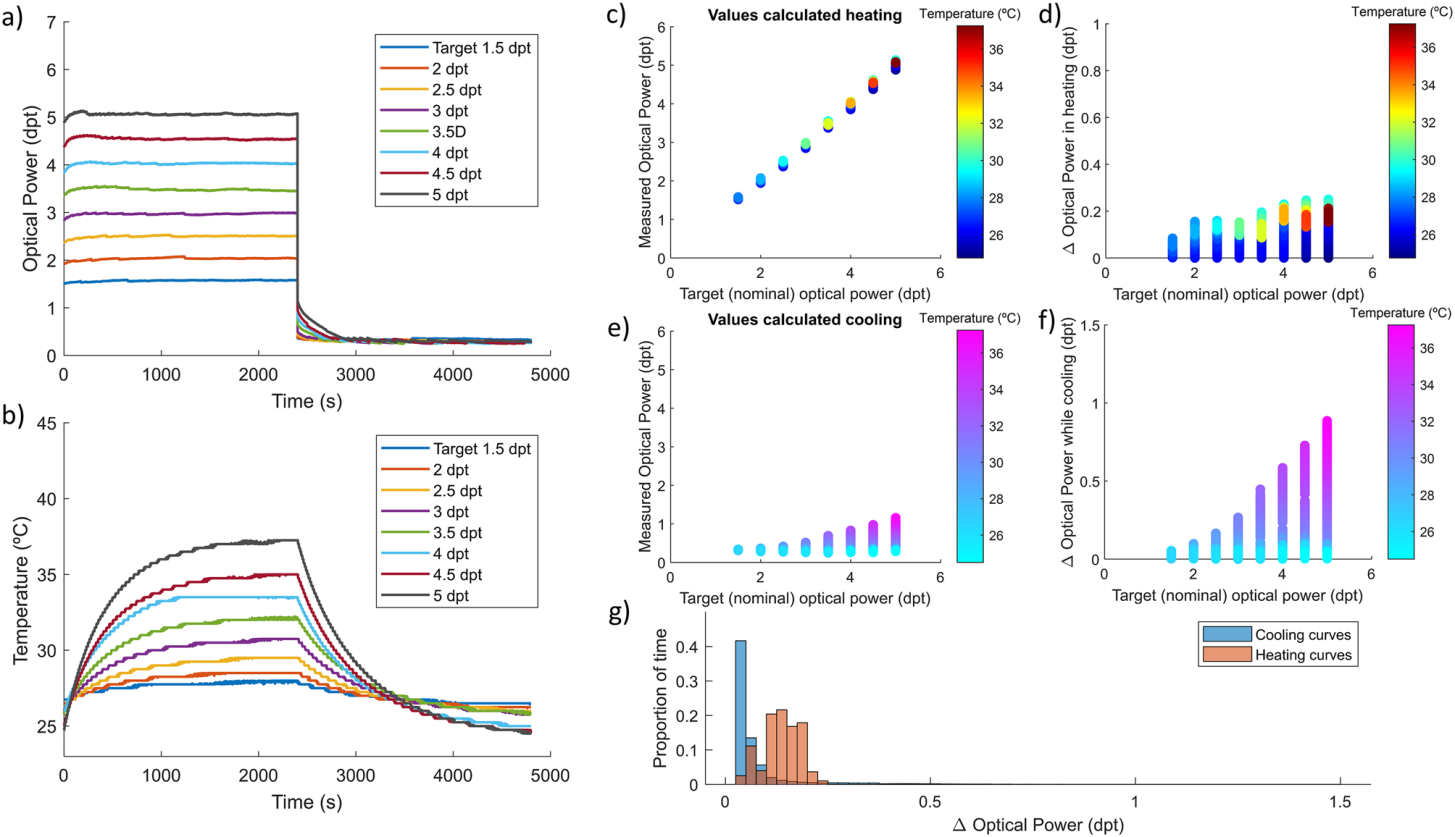
Polynomial model compensation of temperature effect in a TL during long static states of different optical power. (**a**) Graphical description of the result of compensation strategy showing the optical power stabilization in time. (**b**) Internal temperature profile of the lens during the experiment with different target power states. Measured optical power of the lens while heating (**c**) with different driving signals and cooling (**e**) at rest state. Estimated deviation calculated from the difference between target power and measured optical power in the heating (**d**) and cooling (**f**) processes. (**g**) Histogram of optical power deviation while heating (red) and cooling (blue).

Figure 3 summarizes the results of the polynomial model to compensate for temperature variations. In this case, the resting power during the experiment corresponds to 0.00 dpt instead of 1.00 dpt since the equation covers a wider range, and it was interesting to increase the requirements. In this case, the initial temperature is very similar for all the measurements and appears clear in Fig. 3b that the temperature variations are directly proportional to the driving signal. The correction works a little bit worse than in the optimized fit model on the heating curves (Fig. 3a,c). The maximum value is around 0.25 dpt (Fig. 3d) against 0.20 dpt for the optimized fit and 1.00 dpt for non-corrected. Also, in the first part of the cooling curves in Fig. 3a, an important deviation is observed in Fig. 3e, especially when temperature shifts are greater. It is evaluated quantitatively in Fig. 3f with a maximum of 0.9 D but in the histogram (Fig. 3g) is shown that this deviation occurs in a small proportion of time corresponding to the optical power step down. This could be explained by the fact that the home power state (the position in which the lens cools) is closer to 0.00 dpt and is lower than in the previous model. Values around zero give less range in the correction since when we reach the minimum electrical signal we can generate, we cannot compensate further.

**Figure 4:**
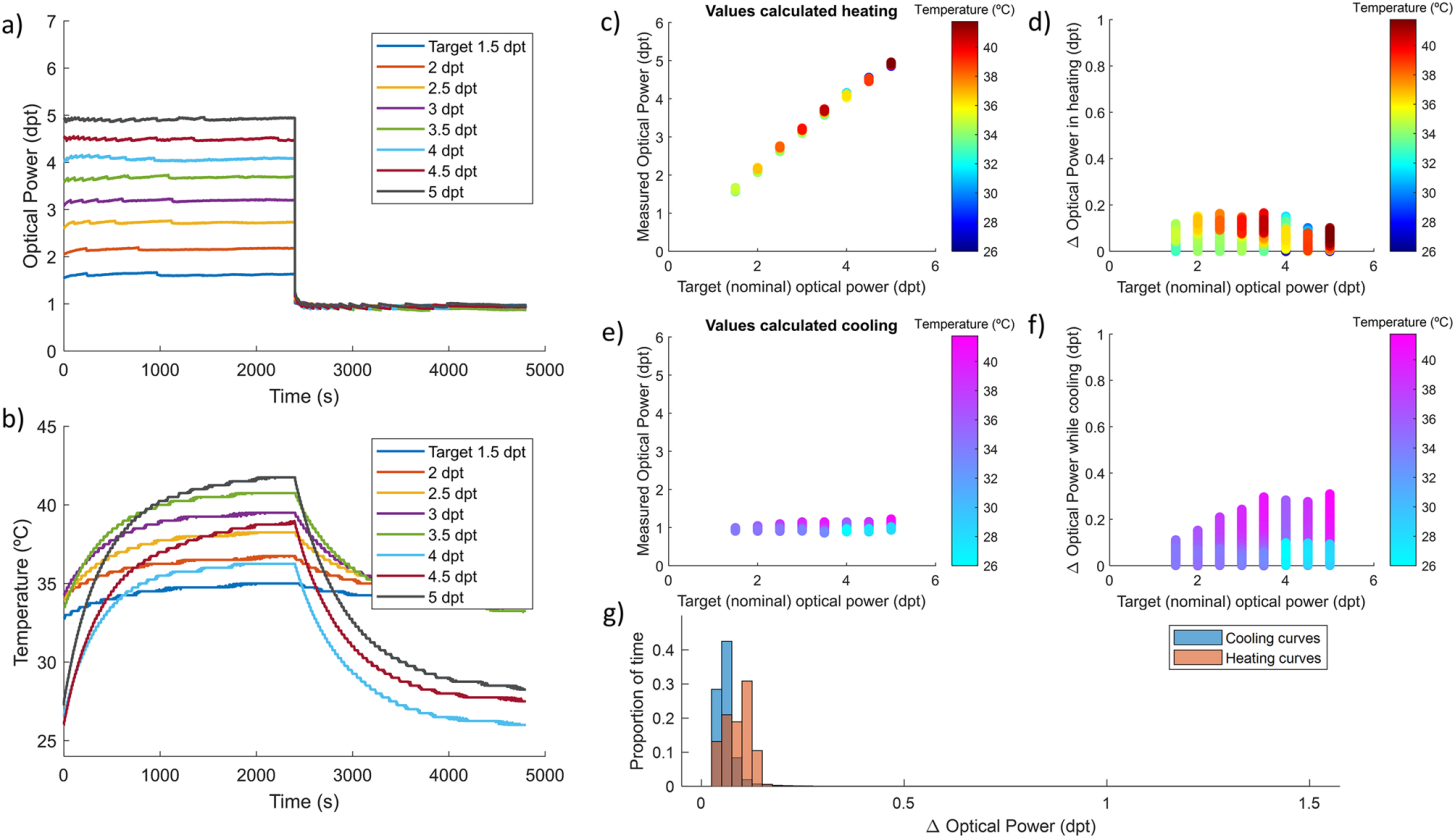
Experimental model compensation of temperature effect in a TL during long static states of different optical power. (**a**) Graphical description of the result of compensation strategy showing the optical power stabilization in time. (**b**) Internal temperature profile of the lens during the experiment with different target power states. Measured optical power of the lens while heating (**c**) with different driving signals and cooling (**e**) at rest state. Estimated deviation calculated from the difference between target power and measured optical power in the heating (**d**) and cooling (**f**) processes. (**g**) Histogram of optical power deviation while heating (red) and cooling (blue).

Figure 4a illustrates the working principle of the temperature compensation algorithm using the experimental model. In the temperature curve, Fig. 4b, we can see that some of the measurements start at different initial temperatures, but the correction strategy is designed to handle this situation and therefore does not affect the outcome. The results of optical power are very steady for the experimental method while the TL is heating (Fig. 4c) as well as while cooling (Fig. 4e). Thus, resulting in less than 0.18 dpt maximum amplitude deviation in the heating process (Fig. 4d) and less than 0.35 dpt in the cooling process (Fig. 4f). Finally, the histogram in Fig. 4g confirms the stability of the results exhibiting less than 0.2 dpt error during most of the experiment. To put into context these values, deviations of less than 0.25 dpt are not corrected in refraction tests in optometry since they are irrelevant to visual acuity.

To confirm the results obtained in the previous experiments performed in one sample lens, and as a repeatability test, a second TL of the same model was used. The characterization procedure was not performed again since the objective was to test if the correction methods could be directly applied to other lenses in a production environment or some adjustments must be done. In this case only polynomial equation and experimental model were used. The results are presented in Table 1.

**Table 1:** Summary results of optical power deviation for the three models and repetition measurements.

Correction method	Maximum ∆OP heating (dpt)	Maximum ∆OP cooling (dpt)
No correction (characterization)	1.00	1.60
Optimized fit	0.16	0.50
Polynomial fit (2lenses)	0.25	0.90
	0.12	0.70
Experimental correction (2lenses)	0.18	0.35
	0.27	0.45

When testing the method in this second lens, we observed that the compensations improve the performance of the lens, and the results are similar to those obtained with the first sample. With the polynomial equation, the maximum deviation is even smaller than for the first lens with 0.12 dpt for heating and 0.7 dpt for cooling versus 0.25 dpt and 0.9 dpt (Table 1). Therefore, the temperature effect seems to be very similar for different samples of the same TL model.

### Hysteresis experiment

**Figure 5:**
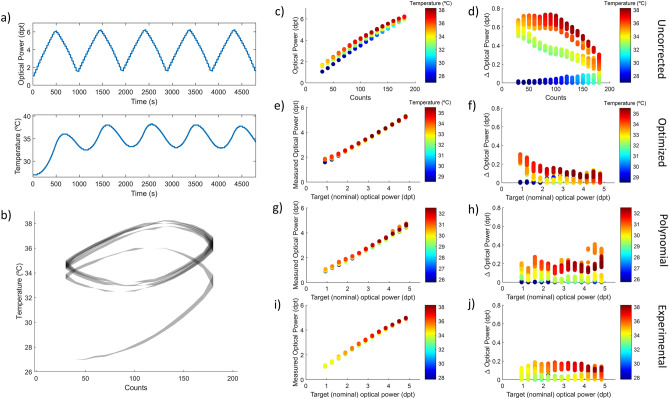
Hysteresis experiment description and performance of the three compensation methods used to correct temperature effect. (**a**) Optical power and temperature profile of the lens during a hysteresis experiment. (**b**) Internal temperature variation and stability region. (**c,d**) Optical power measured values and deviations with respect to nominal value when no temperature correction is applied. (**e,f**) with optimized model for temperature compensation. (**g,h**) with polynomial model compensation and (**i,j**) with experimental model compensation.

The hysteresis experiment was intended to mimic a scanning application using a TL and test how well the correction strategies worked in faster and cyclic power profiles. To do so, a stairs pattern of optical power states was followed, as described graphically in Fig. 5a. Each state is maintained for 30 s while optical power and temperature are registered. It is interesting to see how the temperature increases when the lens is driven at higher currents and decreases when driven at lower currents reaching a stability range that is several degrees higher than the initial ambient temperature Fig. 5b. In Figs. 5c,d are represented the optical power deviations due to differences in the internal temperature of the lens when no compensation method is applied. These are the reference values with a maximum deviation of 0.80 dpt and an important dispersion. The next graphs of Fig. 5 are results of the application of the three correction models: optimized fit (e, f), polynomial fit (g, h) and experimental equation (i, j). If we analyze the graphs from Fig. 5, we can make the following statements:

The optimized fit model seems to be very precise (less than 0.35 dpt deviation) but have some problems in the lower powers near the origin, due to the nature of the equation model. The polynomial equation seems to be a little bit less precise (less than 0.45 D), especially at higher temperatures and optical power values. However, it does not suffer from distortions around 0.00 dpt. Both models require a long characterization process unlike the experimental compensation. The experimental model works also very well in hysteresis conditions, with a deviation smaller than 0.20 dpt. Also, the residuals do not follow a pattern and are quite homogeneous across the optical range of the lens.

### Random experiment

**Figure 6:**
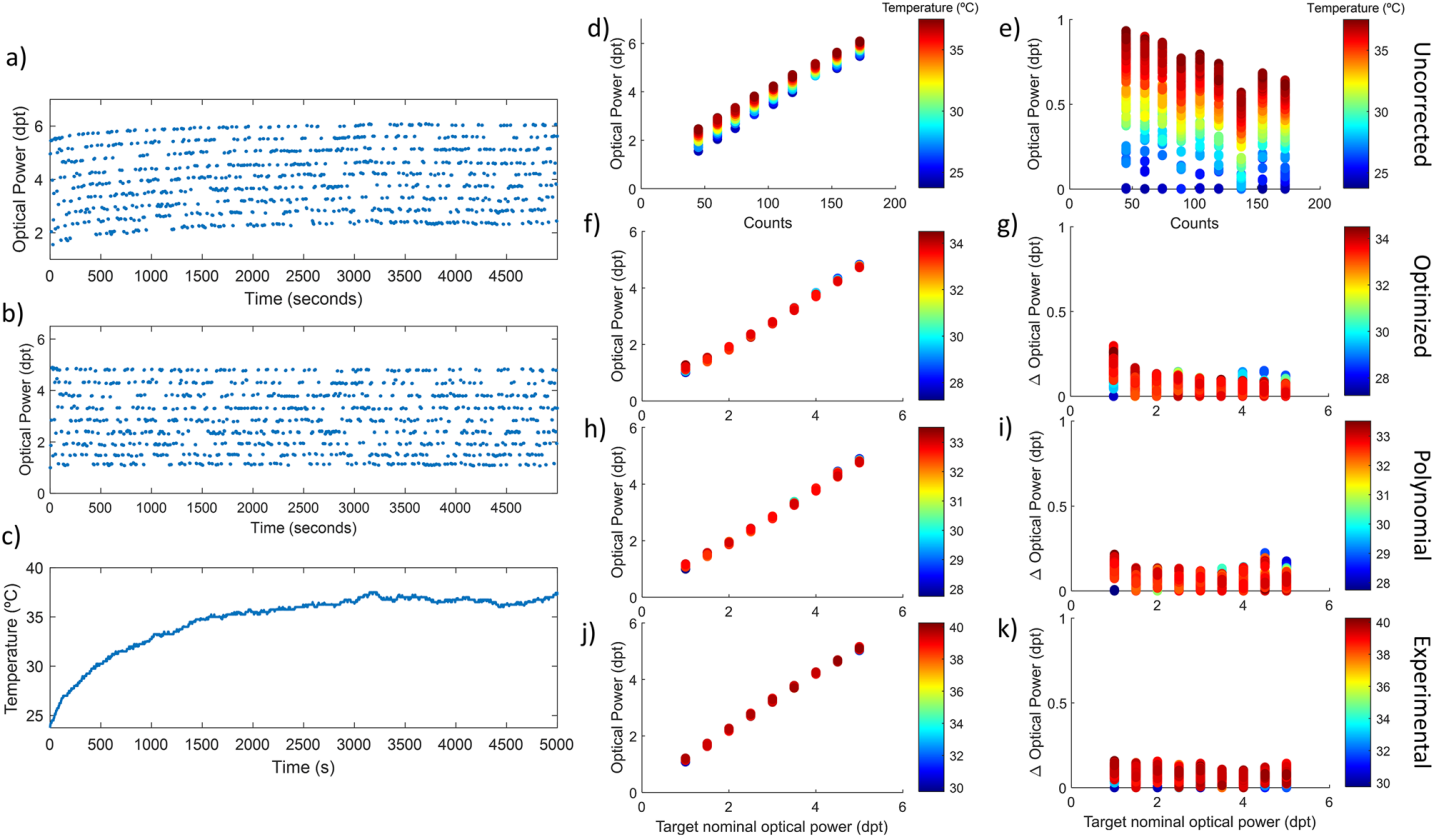
Random experiment description and performance of the three compensation methods used to correct temperature effect. (**a**) Optical power and temperature profile of the lens during a random sequence of power states for characterization without compensation strategy, and (**b**) with the polynomial correction for temperature compensation as an example. (**c**) Internal temperature variation and stability region. (**d,e**) Optical power measured values and deviations with respect to nominal value when no temperature correction is applied. (**f,g**) With optimized model compensation. (**h,i**) With polynomial model compensation. (**j,k**) with experimental model compensation.

In this experiment, instead of applying an up-and-down stair pattern to the lens, it was forced to abruptly and randomly jump between different power states, between 1 and 5 diopters (0.5 dpt step), replicating an exigent situation for the lens as for example, machine vision or 3D laser processing. In Fig. 6a are plotted the optical power measurements for 1000 randomly ordered prefixed points lasting 5 s each, when no temperature compensation is applied. Figure 6b shows the same phenomena but with temperature compensation applied (using the polynomial compensation as an example). If we compare both figures, we can see that the optical power points that correspond to the same driving signal are more stable when the correction is applied and that the distortion due to changes in temperature is less noticeable (Fig. 6c).

Figure 6d and e graphically represent the optical power deviation due to differences in the internal temperature of the lens when characterization is intended, no correction method is applied. These are the reference values and exhibit an important dispersion and a maximum deviation of 1.00 dpt. In Fig. 6f–k the correction models are evaluated: optimized fit (f, g), polynomial fit (h, i) and experimental equation (j, k). The results of the three compensation methods are like the ones obtained in the hysteresis experiments and same conclusions could be extracted: Optimized fit suffers from distortions in low power states. Polynomial is robust and covers a wide range but may have less precision. Finally, experimental show small deviations in the calibration, that can be improved easily, but is more practical for mass characterization and has a robust compensation of the effect of temperature in the whole range of optical power of the TL.

### Dynamic experiments

**Figure 7:**
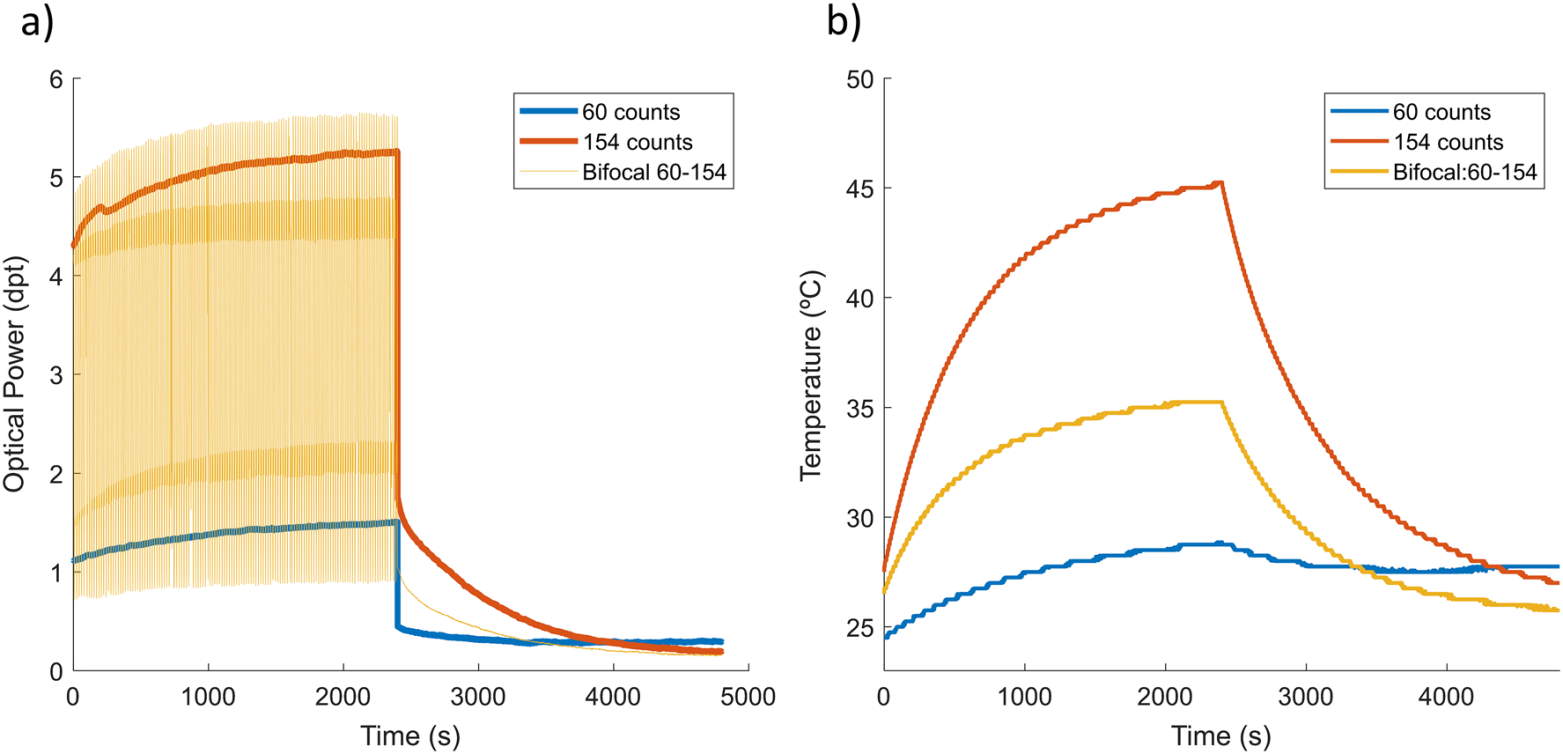
Dynamic experiment description. (**a**) Optical power profile of a static state of 154 counts (red), a low static state of 60 counts (blue) and a bifocal driving signal (yellow) comprising both states. (**b**) Lens internal temperature profile for each signal.

When driving the lens in a dynamic operation, we may expect the internal temperature of the lens to increase further since there is more movement in the liquid and the membrane forming the lens. In this experiment, a bifocal simulation was programmed in the TL and ran for 40 min while registering its internal temperature change. The lens is continuously shifted from a 1.00 dpt state during 10 ms to a 4.00 dpt state during another 10 ms as shown in Fig. 7a. Since the speed of variation of optical power of the lens is faster than the minimum capture period of the camera, it is not possible to register the whole path of the lens. Instead, we are capturing random points that can be used to observe a sample of the optical power points that the lens has gone over. Figure 7b shows the increase in temperature when using the bifocal simulation (yellow curve), the static optical power state of 1dpt (blue curve, 60 digital counts) or the static optical power state of 4dpt (red curve, 154 digital counts) to drive the lens. Bifocal simulation generates an increase in the internal temperature of the lens that is almost equivalent to driving the lens in a static power state corresponding to the weighted mean of the states forming the bifocal simulation.

**Figure 8:**
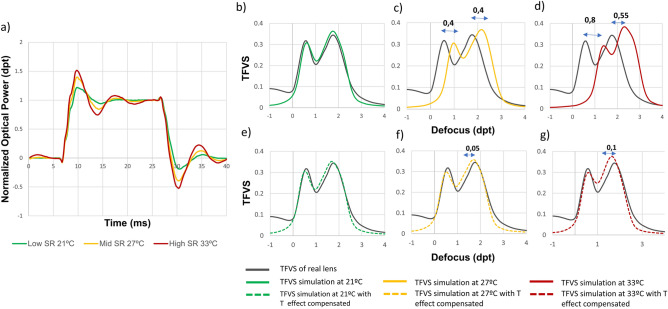
Characterization of TL dynamic behavior at different temperatures. (**a**) Step response measurement of the TL at 3 temperatures 21 °C, 27 °C and 33 °C. TFVS ratio of the simulation of a multifocal lens design at (**b**) 21 °C (**c**) 27 °C and (**d**) 33 °C. The same simulations are performed applying the experimental correction to compensate the deviation in optical power due to temperature (**e**) 21 °C, (**f**) 27 °C and (**g**) 33 °C.

In a second part of the experiment, the step response of the TL was measured at three different temperatures (21, 27 and 33 °C), as illustrated in Fig. 8a. From an overview, the magnitude of the distortions in the transient response seems to increase. The overshoot increases with the internal temperature of the TL. At 21 °C the overshoot has a value of around 25%, while at 33 °C the value increases to 50%. Also, the settling time is greater at higher temperatures going from 10 ms at 21 °C to around 15 ms at 33 °C. To analyze the consequences of the variations in the transient response of the TL due to temperature, a multifocal pattern simulation was measured with internal temperatures of 21 °C, 27 °C and 33 °C. Figure 8b,c and d represent the results of this simulation in terms of through focus visual Strehl ratio metric (TFVS) without applying any compensation strategy. We can observe that as temperature increases, the defocus profile is shifted to higher optical power values showing a deviation comparable to the one observed in the characterization of the TL in static experiments. A maximum of 0.40 dpt deviation at 27 °C and 0.80 dpt at 33 °C. In Fig. 8e,f and g the same simulation is measured but, in this case, the experimental compensation strategy is applied to quantify the performance in fast dynamic situations. A much better accuracy on the simulations is observed. The shift due to the temperature increment and consequently the internal fluid expansion, is compensated. However, there is a slight difference in the shape of the TFVS profile due to the step response variation with temperature.

## Discussion and conclusion

As initially hypothesized, the variations in the internal temperature of the tunable lenses can considerably affect their performance. The main purpose of the experiments carried out was to characterize this phenomenon and to try different methods to correct it to improve the precision of the lenses.

Concerning the experimental characterization, the following statements summarize the results obtained: The internal temperature of the TL depends on the ambient temperature and the electrical signal applied in the previous minutes. The temperature of the lenses increases when driving the lens at medium/high currents. After 40 min at a static nominal power of 5 dpt, the internal temperature exponentially increased from 28 °C to 45 °C. This resulted in the expansion of the internal fluid producing an optical power deviation of 1.0 dpt (1.5 dpt when cooling down), representing a 20% distortion for heating and 30% for cooling. Remarkably, temperature affects more the low-power states when the electrical driving signal is lower.

The characterization of the lens provided enough information to develop three mathematical models as a strategy for compensation of this effect. In case it is not possible to access the internal temperature sensor of the TL, we also developed an equation that can be used to predict the temperature variation due to driving the lens considering only the ambient temperature. To obtain this equation, we have listed all the variables we expected to affect the internal temperature of the lens. The exponential coefficient appeared clear from the shape of the temperature variations and using the Matlab fitting tool, we were able to find a consistent solution. As a result, the heating and cooling process of the TL when driving it at a specific voltage signal can be well described with the following equation:

1 $$T\left(t,{T}_{env},z\right)={T}_{0}+\left(\left(Az+B{z}^{2}\right)+{T}_{env}-{T}_{0}\right)\left(1-{e}^{\frac{-t}{C}}\right),$$

where *T* is the internal temperature of the lens at a certain time, *T*_*env*_ is the environment temperature, *T*_*0*_ is the initial temperature (which can correspond to the environment temperature or any else if the lens has been driven before) and *t* refers to time (in seconds). *A* = 0.02357 and *B* = 0.0005752 are constants that defines the maximum or minimum temperature that the lens can reach depending on the magnitude of the driving signal *z*. Finally, the *C* = 560 constant corresponds to the decay constant of the exponential function and describes the speed at which the temperature reaches the plateau.

**Figure 9:**
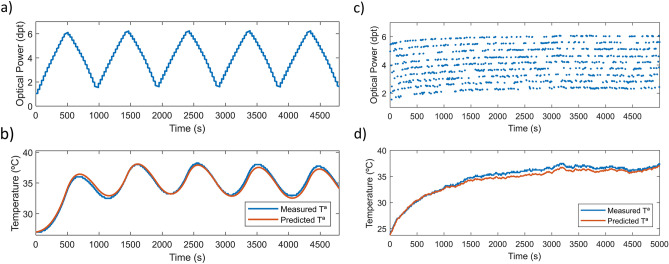
Prediction of internal temperature evolution of a TL in response to different power profiles. (**a**) Measured optical power during hysteresis experiment. (**b**) Measured internal temperature (in blue) and calculated prediction (red) for hysteresis. (**c**) Optical power profile for random experiment and the resulting internal temperature variation (**d**), measured (blue) and predicted (red).

The internal cooling of the lens can be approximated by the same exponential decay equation, similar to the discharge of a capacitor. The sign of the exponential factor is defined by the $((Ax + Bx^{2} ) + T_{env} - T_{0} )$ multiplying term. Each time, a different electrical signal is applied to the TL, the previous equation represents the expected temperature evolution of the lens depending on the signal applied to the lens and the ambient temperature. To show the application of this principle and test the accuracy of the equation, it is applied on the hysteresis and random experiments. Plotted in red in Fig. 9b,d we can observe the predicted temperature variation expected for the lens when driven with a specific signal profile, in this case, following the hysteresis (Fig. 9a) and random (Fig. 9c) experiments.

In terms of temperature, a multifocal simulation (fast alternance among optical powers in cycles of 20 ms) is equivalent to driving the lens with a static power state that corresponds to the mean of all the power states covered by the lens weighted by the time spent at each. This confirms that the internal temperature of the lens is not affected by driving the lens dynamically but by the total amount of current flowing through it.

Also, we found an unexpected result in the transient temporal response of the TL when driven at high speed: the temporal distortions increase with the internal temperature. This phenomenon, not described earlier and only noticeable in fast applications, could be related to potential changes with temperature in the viscosity of the fluid, the elasticity of the membrane, or the mechanism of the actuator.

When the compensations were applied, the lens required less current to achieve the target optical power states. Since the temperature increase depends directly on the applied current, the increase in internal temperature was lower, making the system more stable and, at the same time, reducing the energy consumption.

All the compensation methods described (optimized, polynomial and experimental fitting) provided an improvement in the performance of the lenses. The polynomial equation gives a little worse result in some experiments/conditions than the optimized fit. However, it describes better the behavior of the lens in the complete optical range, especially near 0.00 dpt. The experimental method gives similar results, or even better in some conditions. This is an important result with practical implications, since the last method provides a good calibration without a complete characterization of the lens. Modelling and compensation reduced the maximum deviations from 1.00 to 0.20 dpt when heating (from 1.5 to 0.35 dpt when cooling) and the distortions from 20 to 4% and from 30 to 7% with regard to the 5 dpt maximum nominal step. Compensation reduced temperature effects by more than 75%, representing a considerable improvement in the performance of the lens. Similar improvements were obtained in dynamic and hysteresis experiments. The only drawback of the experimental model is that it presents small deviations in reaching the target nominal power values showing a slight curvature in the relation between measured power against target nominal power in Fig. 6j, for example. This is because the mathematical equation of the experimental model was built with the hypothesis that voltage signal affects the optical power of the TL linearly. However, after analyzing the results of the different experiments, it appears that it could work even better with a second order term.

The correction methods are not influenced by external temperature changes. The ambient temperature was not actively controlled and even if it varied during the experiments (e.g. Fig. 4b), the final optical power was not affected. The compensation models are robust. The same calibration was used in a second lens unit obtaining very similar results. Therefore, it is not necessary to characterize individually each lens. Although it would be the ideal approach to at least quantify the optical power offset of each lens.

In this study, we presented: First a characterization method to evaluate the magnitude of the optical power deviations due to internal temperature shifts in liquid tunable lenses when driven at different operation conditions as static powers, hysteresis cycles, random states and fast dynamic simulations. Second, three different mathematical models (experimental, polynomial and optimized) to predict and correct the response of the lens with temperature, reducing by 75% the deviation in optical power produced. Third, a prediction of the internal temperature shifts while using the TL at different time spans ranging from milliseconds to minutes without accessing the internal temperature sensor. And finally, a real time compensation tool based on the integration of the mathematical model into a custom voltage electronic driver.

## Methods

Static power variations, hysteresis measurements, random optical power variations and dynamic states experiments were carried out to obtain a full characterization of temperature effect on TLs. Three mathematical models were developed to compensate for (and predict) power deviations due to temperature variations. A custom electronic driver of the lens was developed to perform those experiments. To accurately measure the response in optical power of the TLs during the temperature changes, a low-cost low-speed version of a focimetry system^32^ was used.

### Samples and specifications

The experiments in this study were carried out using two units of EL-10-30-C tunable lens model from Optotune AG (Switzerland). The optical power ranges from 5 to 12 diopters (dpt). According to the technical specifications of the TL model^29^, internal temperature variations have two effects: When heating up, first, the refractive index of the fluid decreases, producing a decrease in optical power. Second, the internal fluid of the lens expands in volume ending up in an increase of the optical power. The second effect prevails and the resulting increase in optical power has been evaluated to be approximately 0.7 diopters for a 10 °C temperature increase. Also, according to the TL manufacturer, the sensitivity in optical power to temperature changes along the dioptric range. The sensitivity is higher (around 10%) when the lens is driven at low currents, and lower when the lens is driven close to its higher current limit. The study was performed using two samples of one specific lens model, but the methods and strategies we are presenting can be applied to other TLs that have similar working principles and suffer from temperature variation effects.

### High-speed tunable lens driver and temperature registering

In previous works from Dorronsoro et al.^32^, a custom high-speed driver for TLs was specifically developed for temporal multiplexing applications. It includes hardware, firmware and a control interface. The electronics basis comprises an Arduino Nano 3 (Arduino, Italy) and a DRV8833 Dual Motor Driver (Texas Instruments, TX). The lens is driven with a voltage pulse width modulation (PWM) signal at 32 kHz to achieve a high temporal accuracy for generating fast dynamic optical power profiles (in the order of milliseconds; ms). It can also work in dual channel, with synchronization, for binocular applications. The amplitude of the PWM voltage signal ranges from 0 to 5 V and is specified in digital counts that go from 0 to 255 (an 8-bit timer is used to generate the signal). The ‘digital counts’ notation is used in the calibration of the lens and during the experiments. The reason for using it is because exact voltage values would require an additional voltmeter constantly plugged in and metric conversion to interact with the driver, thus increasing unnecessarily the computation requirements when applying the correction models presented in Sect.  “Mathematical models and compensation strategies”. The custom driver fulfills all the compatibility requirements of the TL model used in this study. Also, it uses the temperature sensor included (SE97B) to register internal temperature data with 0.125 °C resolution. The firmware is configured to retrieve the internal temperature of the TLs each half second using the I2C protocol. The control interface is developed in Matlab 2019 (MathWorks, Natick, MA) and has the capability to drive the tunable lens in different modes and display its internal temperature as well as some flow monitoring messages. Ambient temperature was not actively controlled during the experiments since in previous tests we observed that at room temperature (10 °C to 35 °C) it only affected the initial internal temperature of the lens but not its behavior. The results of prediction of internal temperature discussed in Eq. (1) confirm this statement.

### Measurement system

To estimate the TL’s optical power, we used a custom-built optical set-up that converts the optical powers of the TL into lateral displacements of a reference object. The concept was previously described as High-Speed Focimetry^33^, but in this study we use a low-cost implementation with a conventional CMOS camera (DCC1545 by Thorlabs, Inc. US) instead of a high-speed camera, and a leaking optical fiber (chemically attacked with acetone, coupled to a red laser), as a reference object instead of an illuminated slit. The lateral shift of the reference, that gauges the optical power, is generated with a prism. The images are processed using custom Matlab routines, applying the results of a calibration performed a-priori (see “Calibration of measurement system and driver”). The TL is mounted in an optical module that reduces the offset optical power of the lens by 6 diopters to bring the minimum power close to zero diopters. Also, it makes the system more compact, encapsulating the sample lens in a plastic case that will retain heat, simulating the use of the TL in real world applications.

### Calibration of measurement system and driver

First, a direct relationship between the lateral displacement of the stimulus image (in pixels) and the optical power (in diopters, dpt) is obtained by using trial lenses, from -1D to + 5D, at a secondary pupil plane optically conjugated to the TL under study, while the TL is at idle state (no current). Once the measurement system is characterized, a calibration of the driver and TL set is performed without any trial lens in the system to obtain a first estimation of the relation between voltage applied to the lens (in digital counts from 0 to 160 counts) and the position of the stimulus (in pixels). The precision of the system is 0.05 dpt which corresponds to 1 pixel displacement of the stimulus image. Those measurements are performed without considering the effect of temperature on the lens, although the internal temperature of the TL was maintained stable during the whole process by including a rest time of 20 s between each digital count measurement. Then by using both calibrations (system + driver), the relationship between the voltage applied to the lens (in digital counts) and the optical power it provides (in diopters) is obtained.

### Experiments to characterize the effect of the temperature on the tunable lens

We performed four sets of experiments to characterize the effect of the variations in internal temperature on the optical power of the TL, in the complete range of optical power and in different situations, to guide, adjust, and evaluate the compensation strategies introduced in Sect. “Mathematical models and compensation strategies”.

#### Static experiment

In this first experiment the lens was driven with a predefined voltage signal for 40 min and then with a home value (corresponding to 1 dpt) for other 40 min. Nine predefined voltage signals were measured, corresponding to optical powers from 1.50 to 5 dpt, in 0.50 dpt steps. The whole experiment took 12 h per lens, providing a complete and precise characterization of the internal temperature variations and the lens behavior during heating and cooling. The results of this experiment were used to adjust the mathematical models guiding the compensation of temperature effects, described in Sect. “Mathematical models and compensation strategies”. This is the simplest scenario in which we could use a TL lens. Maintaining a certain optical power during seconds or minutes while performing another task with the optical system, as in a traditional microscope or in a photography session after finding the right focus.

#### Hysteresis experiment

In this second experiment, the TL input was a stair of states (digital counts), starting from 30 counts and ending at 180 counts in steps of 10, then a descending from 180 to 30, to close the cycle. Each state was driven during 30 s in which the internal temperature and optical power are registered every 0.5 s. Each cycle was repeated 5 times, to test the hysteresis of the TL. This experiment mimics the behavior of the TL in slow scanning applications as 3D microscopy.

#### Random experiment

To perform the third experiment, an array of 1000 randomly ordered optical states, corresponding to optical powers in between 1 and 5 dpt (in steps of 0.50 dpt) was created according to the initial calibration. Each power state was maintained for 5 s while the internal temperature was registered. Here, the hysteresis is tested in a different way, making the lens work at random steps to analyze the behavior across the whole range and for different magnitude jumps.

#### Dynamic states experiment and step response

In some applications, as in temporal multiplexing for the simulations of multifocal corrections, or in-depth sectioning of a volume, it is necessary to change the optical power of the tunable lens every few milliseconds, with abrupt transitions. The dynamic behavior of the lens is critical in several applications as fast scanning or multifocal ophthalmic correction simulations, as well as the drift induced by internal temperature variations.

In the first part of this last experiment, a bifocal simulation (a bistate wave of nominal optical power shifting between 1 and 4 dpt every 10 ms) was programmed and ran for 40 min while the internal temperature changes were registered. The results are compared to a monofocal simulation (a stable state).

The second part of this fourth experiment consisted in measuring with a high-speed camera^32^, at three internal temperatures (21, 27 and 33 °C) that are expected to be reached during operation of the lens at room temperature: (1) the step response describing the dynamic behavior of the TL; and (2) a complex multifocal pattern simulation very sensitive to the shape of the step response^34^. The multifocal simulation is very similar to the bifocal simulation but have different additions or optical power coefficients and the electrical signal is optimized to compensate the transient response of the lens measured at 21 °C^35^. The performance of the TL simulation at different temperatures was evaluated in terms of the through-focus Visual Strehl ratio (TFVS)^36^, and compared with the real physical lens simulated.

### Mathematical models and compensation strategies

It is important to mention that the manufacturer have developed a custom method to compensate the temperature effects^37^. It is based on an interpolation algorithm that makes use of an internally stored calibration of the lens’ optical power versus current at two temperatures (20 °C and 50 °C). The results are very good (0.1 dpt maximum deviation) but require using a specific current driver that limits the design possibilities. For example, driving the lens with a voltage signal, binocular applications, or the need of using the TL integrated in an optical system requiring a specific characterization. For these reasons, more robust mathematical models of the behavior of the tunable lens, in a 3-dimensional space of optical power, internal temperature, and electrical signal, were developed and used to guide strategies to characterize, compensate and predict the effect of temperature.

**Figure 10:**
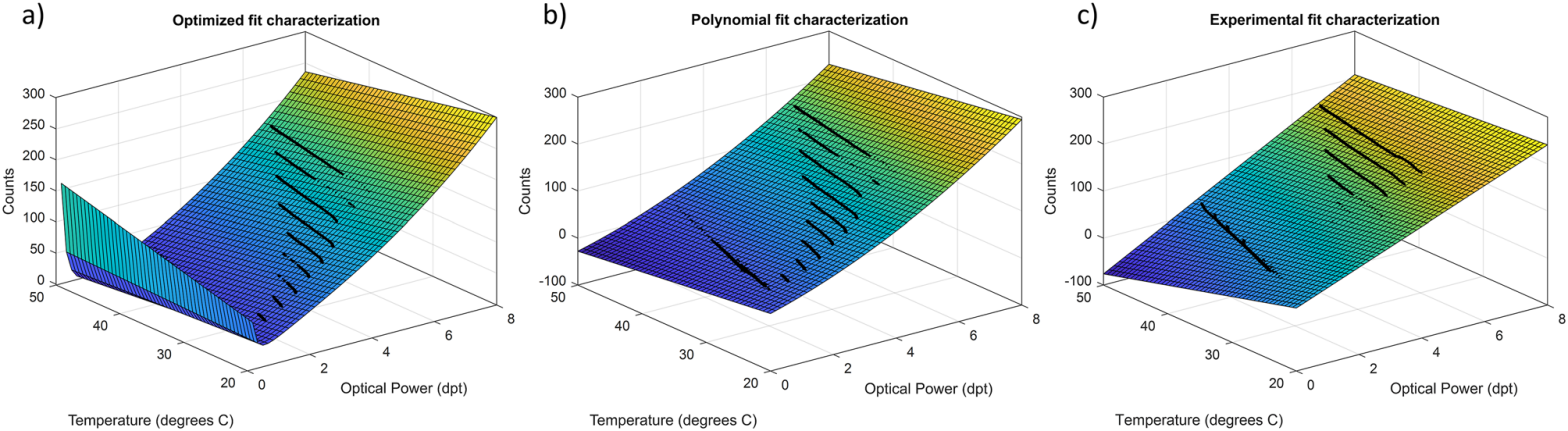
Graphical representation of the 3D fitting models tested as a compensation method for temperature effects on the TL. Optimized model (**a**), Polynomial model (**b**), and experimental model (**c**).

Analyzing the experiments, it is clear, at least on the characterization using the long heating and cooling cycles of alternating static powers (Sect. “Static experiment”), that the dependent variable is the optical power, while the independent ones are the voltage driving the lens and its temperature. However, the model equations are written with driving voltage as the dependent variable since it is an inverse regression or what is called in statistics, a calibration problem^38^. We are trying to estimate the driving electrical signal required to achieve a target optical power. In this way, the correction equations can be directly implemented on the electronic driver to test their accuracy. We tested many non-linear equations using the Trust-Region algorithm of the Matlab’s curve fitting tool, which were evaluated considering the goodness of the fit to the characterization experiment data. Three models were finally developed and are referred to as the optimized, the polynomial and the experimental. They are built on the same basis: an offset constant and two linear coefficients, one relating target optical power to the voltage signal, and the other relating temperature to the electrical signal. Starting from this foundation, additional terms were included to try to improve the goodness fitting indicators following different strategies. Figure 10 shows the graphical representation of the three models developed.

#### Optimized model

In the optimized model, several types of coefficients were tested, including exponentials, square roots, and different kind of cross terms between temperature and optical power with just the objective to find the best fitting performance while maintaining the possibility to include the calculations on a simple microcontroller. The resulting equation is the following:

2 $$Counts\left(x,y\right)= A + B*x + C*{x}^{2} + D*y + E*\frac{y}{x} + F*\sqrt{x},$$

where *x* corresponds to optical power in dpt and *y* corresponds to the temperature of the lens (ºC). The 3D curve representation is illustrated in Fig. 10a. The coefficients providing the best fit were: *A* = − 23.760, *B* = − 19.230, *C* = 3.338, *D* = − 2.265, *E* = 0.852 and *F* = 108.900.

As can be observed in Fig. 10, the optimized model is diverging near 0 dpt which is not a real description of the lens physical behavior. This is because there is no characterization data at this part of the optical range. However, in the implementation of the algorithm in the microcontroller the lens is limited to not use this part of the range. The temperature correction algorithm reduces temperature effect when the lens is warmer by decreasing the electrical signal, therefore near zero diopter conditions can only be reached at certain temperatures (due to physical limitation of the lens).

To compare the models, three different standard indicators evaluating the goodness of the fitting were used: Sum of squared estimate of errors (SSE), Coefficient of determination (R-square) and Root mean square error (RMSE). The number of samples used to build the three models is 9600. For the Optimized model the results were: SSE 5.167 × 10^4^, R-square 0.9997, and RMSE 0.794. To make easier the evaluation of the fitting factor results, 1 count with the lens at 30 °C can be approximated to an average value of 0.034 dpt according to the characterization dataset.

#### Polynomial model

The Polynomial model, illustrated in Fig. 10b, followed a second-order polynomial equation in optical power and a first-order polynomial in temperature, with a first-order cross term:

3 $$Counts\left(x,y\right)= A + B*x + C*y + D*{x}^{2} + E*x*y,$$

where *x* corresponds to optical power in dpt, and *y* refers to the temperature of the lens in °C. The coefficients obtained were: *A* = 55.990, B = 20.100, C = − 1.676, D = 1.904 and E = − 0.064, and the goodness parameters SSE 6.921 × 10^4^, R-square 0.9996 and RMSE 0.919. If added, other polynomial terms result in non-relevant coefficients (< 10^–5^), inducing confusing dependencies without improving the goodness of the fit.

Even though the polynomial model provided worse fit indicators, it resulted in a better representation at low optical powers. Also, it is simpler physically and requires less computational resource. While the polynomial model represents near 0 dpt values with a lower electrical signal, the optimized model equation diverges near 0 due to the $\frac{y}{x}$ coefficient.

#### Experimental model

The model describing the experimental method (Fig. 10c) is based on the simple calibration of the driver presented in Sect. “Calibration of measurement system and driver”: a linear equation relating optical power and voltage, obtained at a reference temperature. To compensate the effects of temperature, two components were added in the equation. The first one, *C*, is a fixed coefficient that introduces a linear dependence with the temperature increment, and the second one, *D*, introduces a cross term between temperature increment and target optical power. This last coefficient was intended to compensate for the distinct sensitivity of the lens to temperature across its optical power range. This phenomenon was described by the manufacturer and observed in the initial characterizations. The value of the coefficient and its sign was fixed using an experimental optimization process. The resulting equation is the following:

4 $$Counts\left(x,y\right)= A + B*x - \left(y - {T}_{C}\right)* \left( C + D*\left(1 -\frac{x+1}{{x}_{max}}\right)\right),$$

where *x* corresponds to optical power in dpt, *y* refers to the actual temperature of the lens in °C, ${T}_{C}$ is the reference temperature at which the calibration was performed and *x*_*max*_ is the maximum value of optical power of the lens. The coefficients *A* and *B* are defined by the calibration, in this case *A* = 2.880 and *B* = 30.310. The compensation coefficients were optimized considering the electrical driver resolution and the smallest variation in temperature producing a noticeable deviation in optical power, resulting in optimal values *C* = 0.500 and *D* = 1.500. This model provides a SSE 2.234e + 06, a R-square 0.988 and a RMSE: 5.223.

#### Implementation of compensation strategy in TL driver

Once the model equations were fitted, the compensation strategy was implemented in the TL driver by storing the equation in the memory of the microcontroller. The algorithm developed, checks the temperature every 0.5 s. Each time the temperature increases or decreases 1 °C or more, the voltage driving the lens is recalculated according to the equation and applied to compensate the temperature effects. The temporal sampling period was chosen to correctly trace the temperature variations without overcharging the microcontroller. The temperature variation limit was set considering the resolution of the timer (8 bits) generating the PWM voltage signal, to avoid unnecessary calculations providing variations below the rounding threshold.

## Data Availability

Data underlying the results presented in this paper are not publicly available at this time but may be obtained from the authors upon reasonable request. Please contact Y.M. at ymarrakchi@2eyesvision.com for more information.

## References

[1] Blum, M., Büeler, M., Grätzel, C. & Aschwanden, M. Compact optical design solutions using focus tunable lenses. In *Proc. SPIE 8167*, Optical Design and Engineering IV, 81670W. 10.1117/12.897608 (2011).

[2] Casutt, S., Bueeler, M., Blum, M. & Aschwanden, M. Fast and precise continuous focusing with focus tunable lenses. In *Proc. SPIE 8982*, Optical Components and Materials XI, 89820Y. 10.1117/12.2037516 (2014).

[3] Bathe-Peters M, Annibale P, Lohse MJ (2018). All-optical microscope autofocus based on an electrically tunable lens and a totally internally reflected IR laser. Opt. Express.

[4] Li H, Cheng X, Hao Q (2015). An electrically tunable zoom system using liquid lenses. Sensors.

[5] Zou Y, Chau FS, Zhou G (2017). Ultra-compact optical zoom endoscope using solid tunable lenses. Opt. Express.

[6] Volpi D (2017). Electrically tunable fluidic lens imaging system for laparoscopic fluorescence-guided surgery. Biomed. Opt. Express.

[7] Grulkowski I, Manzanera S, Cwiklinski L, Sobczuk F, Karnowski K, Artal P (2018). Swept source optical coherence tomography and tunable lens technology for comprehensive imaging and biometry of the whole eye. Optica.

[8] Fuh Y-K, Chen J-K, Chen P-W (2015). Characterization of electrically tunable liquid lens and adaptive optics for aberration correction. Optik.

[9] Vinas M (2019). Visual simulators replicate vision with multifocal lenses. Sci. Rep..

[10] Kim C-S, Kim W, Lee K, Yoo H (2019). High-speed color three-dimensional measurement based on parallel confocal detection with a focus tunable lens. Opt. Express.

[11] Chong C (2020). Four-dimensional visualization of zebrafish cardiovascular and vessel dynamics by a structured illumination microscope with electrically tunable lens. Biomed. Opt. Express.

[12] Shi R (2019). Multi-plane, wide-field fluorescent microscopy for biodynamic imaging in vivo. Biomed. Opt. Express.

[13] Léonard J, Lee M, Morales A, Karg TM, Esslinger T, Donner T (2014). Optical transport and manipulation of an ultracold atomic cloud using focus-tunable lenses. New J. Phys..

[14] Falleroni F, Torre V, Cojoc D (2018). Cell mechanotransduction with piconewton forces applied by optical tweezers. Front. Cell. Neurosci..

[15] Jian Z, Tong Z, Ma Y, Wang M, Jia S, Chen X (2020). Laser beam modulation with a fast focus tunable lens for speckle reduction in laser projection displays. Opt. Lasers Eng..

[16] Konrad, R., Cooper, E. A., Wetzstein, G. Novel optical configurations for virtual reality: evaluating user preference and performance with focus-tunable and monovision near-eye displays. In *Proc. of the 2016 CHI Conference on Human Factors in Computing Systems* 1211–1220 10.1145/2858036.2858140 (2016).

[17] Lin H-C, Chen M-S, Lin Y-H (2011). A review of electrically tunable focusing liquid crystal lenses. Trans. Electr. Electron. Mater..

[18] Berge B, Peseux J (2000). Variable focal lens controlled by an external voltage: An application of electrowetting. Eur. Phys. J. E.

[19] Chen Q, Li T, Zhu Y, Yu W, Zhang X (2018). Dielectrophoresis-actuated in-plane optofluidic lens with tunability of focal length from negative to positive. Opt. Express.

[20] Dong L, Agarwal AK, Beebe DJ, Jiang H (2006). Adaptive liquid microlenses activated by stimuli-responsive hydrogels. Nature.

[21] Moran PM, Dharmatilleke S, Khaw AH, Tan KW, Chan ML, Rodriguez I (2006). Fluidic lenses with variable focal length. Appl. Phys. Lett..

[22] Mao X, Lin S-CS, Lapsley MI, Shi J, Juluri BK, Huang TJ (2009). Tunable liquid gradient refractive index (L-GRIN) lens with two degrees of freedom. Lab Chip.

[23] Bernet S, Harm W, Ritsch-Marte M (2013). Demonstration of focus-tunable diffractive Moiré-lenses. Opt. Express.

[24] Han Z, Colburn S, Majumdar A, Böhringer KF (2020). MEMS-actuated metasurface Alvarez lens. Microsyst. Nanoeng..

[25] Fahrbach FO, Voigt FF, Schmid B, Helmchen F, Huisken J (2013). Rapid 3D light-sheet microscopy with a tunable lens. Opt. Express.

[26] Akondi V, Dorronsoro C, Gambra E, Marcos S (2017). Temporal multiplexing to simulate multifocal intraocular lenses: Theoretical considerations. Biomed. Opt. Express.

[27] Ashtiani AO, Jiang H (2013). Thermally actuated tunable liquid microlens with sub-second response time. Appl. Phys. Lett..

[28] Zhang H, Ren H, Xu S, Wu S.-T. (2014). Temperature effects on dielectric liquid lenses. Opt. Express.

[29] Optotune, A.G. Fast electrically tunable lens EL-10–30 series.

[30] Kawashima, Y. Liquid lens with temperature compensated focus time, US20110200314A1. https://patentimages.storage.googleapis.com/89/31/d2/60ee04f4468540/US20110200314A1.pdf (Accessed 24 March 2011).

[31] Patscheider, R., Niedere, D., Gebbers, P., Borer, D., Laning, C., Smolka, S. Temperature drift compensation for liquid lenses, US20180136372A1. https://patents.google.com/patent/US20180136372A1/en (Accessed 17 June 2016).

[32] Dorronsoro C (2019). Tunable lenses: Dynamic characterization and fine-tuned control for high-speed applications. Opt. Express.

[33] Dorronsoro, C., Gambra, E., Barcala, X., Rodríguez, V., Marcos, S. Device for determining the optical power of lenses and measurement method, WO/2019/002656. https://patentscope.wipo.int/search/en/detail.jsf?docId=WO2019002656&recNum=1&office=&queryString=carlos+dorronsoro&prevFilter=&sortOption=Pub+Date+Desc&maxRec=77 (Accessed 28 June 2018).

[34] Vinas M (2020). Optical and visual quality with physical and visually simulated presbyopic multifocal contact lenses. Transl. Vis. Sci. Technol..

[35] Akondi V, Sawides L, Marrakchi Y, Gambra E, Marcos S, Dorronsoro C (2018). Experimental validations of a tunable-lens-based visual demonstrator of multifocal corrections. Biomed. Opt. Express.

[36] Iskander DR (2006). Computational aspects of the visual strehl ratio. Optom. Vis. Sci..

[37] Optotune, A.G. Electrical Lens Driver 4, Optotune AG, Switzerland, Datasheet. https://www.stemmer-imaging.com/media/uploads/software/10/102968-Optotune-LensDriver4-Manual.pdf (Accessed June 2019).

[38] Upton GJG, Cook I (2008). A Dictionary of Statistics.

